# TATA-Like Boxes in RNA Polymerase III Promoters: Requirements for Nucleotide Sequences

**DOI:** 10.3390/ijms21103706

**Published:** 2020-05-25

**Authors:** Karina A. Tatosyan, Danil V. Stasenko, Anastasia P. Koval, Irina K. Gogolevskaya, Dmitri A. Kramerov

**Affiliations:** Laboratory of Eukaryotic Genome Evolution, Engelhardt Institute of Molecular Biology, Russian Academy of Sciences, 119991 Moscow, Russia; tatkarik@gmail.com (K.A.T.); stasenkodanilvladimirovich@gmail.com (D.V.S.); panikoval@yandex.ru (A.P.K.); irina_imb@inbox.ru (I.K.G.)

**Keywords:** RNA polymerase III, promotor, TATA box, transcription factor, ncRNA gene, SINE

## Abstract

tRNA and some other non-coding RNA genes are transcribed by RNA polymerase III (pol III), due to the presence of intragenic promoter, consisting of boxes A and B spaced by 30–40 bp. Such pol III promoters, called type 2, are also intrinsic to Short Interspersed Elements (SINEs). The contribution of 5′-flanking sequences to the transcription efficiency of genes containing type 2 promoters is still studied insufficiently. Here, we studied this issue, focusing on the genes of two small non-coding RNAs (4.5SH and 4.5SI), as well as B1 and B2 SINEs from the mouse genome. We found that the regions from position −31 to −24 may significantly influence the transcription of genes and SINEs. We studied the influence of nucleotide substitutions in these sites, representing TATA-like boxes, on transcription of 4.5SH and 4.5SI RNA genes. As a rule, the substitutions of A and T to G or C reduced the transcription level, although the replacement of C with A also lowered it. In 4.5SH gene, five distal nucleotides of −31/−24 box (TTCAAGTA) appeared to be the most important, while in the box −31/−24 of 4.5SI gene (CTACATGA), all nucleotides, except for the first one, contributed significantly to the transcription efficiency. Random sequences occurring at positions −31/−24 upstream of SINE copies integrated into genome, promoted their transcription with different efficacy. In the 5′-flanking sequences of 4.5SH and 4.5SI RNA genes, the recognition sites of CREB, C/EBP, and Sp1 factors were found, and their deletion decreased the transcription.

## 1. Introduction

Unlike RNA polymerase II (pol II), which transcribes both protein-coding genes and non-coding genes, RNA polymerase III (pol III) synthesizes only relatively short non-coding RNAs (ncRNAs). However, among pol III transcripts, there are a lot of RNAs crucial for eukaryotic cell activity [1,2]. For example, 7SK RNA is a transcription regulator of a number of protein-coding genes, U6 RNA is involved in mRNA splicing, whereas tRNA, 5S rRNA, and 7SL RNA are necessary for translation, or engaged in its regulation. Y RNAs take part in the initiation of DNA replication [3], RPR and MRP are required for processing of pre-tRNAs and pre-rRNAs, respectively, whereas vtRNA acts as a riboregulator of cellular autophagy process [4]. In addition to these RNAs, which are characteristic of all or vast majority of eukaryotes, apparently, a lot of pol III transcripts exist, which are common only among specific phylogenetic groups. The functions of some of them are already determined: for example, BC1 RNA and BC200 RNA are involved in translation regulation in the neurons of rodents and anthropoids, respectively [5]. Some human pre-miRNAs are synthesized by pol III [6], and obviously such lineage-specific pol III transcribed miRNA genes exist in other higher eukaryotes as well. A number of other ncRNAs transcribed by pol III from specific regions of human genome were described [7]; one of such RNAs is apparently involved in regulation of cell proliferation [8].

There are at least three types of promoters for pol III. Type 3, which was identified most recently, resembles the promoter for pol II, i.e., it is entirely located in the 5′-flanking sequence (5′-FS) [1,9]. This type of pol III promoter is characteristic of the following RNA genes: U6, 7SK, Y, RPR, MRP, and selenocysteine tRNA. Type 3 promoters consist of: (i) canonical TATA box located near −30 position upstream of the transcription start site (TSS); (ii) the proximal sequence element (PSE) at positions −50 to −60; and (iii) the distal sequence element (DSE), which is located even further from the TSS (usually around positions −210/−240). DSE contains an SPH-element, which binds the STAF transcription factor, or an octamer sequence, which recruits the Oct-1 transcription factor. PSE contains a specific nucleotide sequence recognized by the SNAPc protein complex, which consists of five different subunits. TATA box recruits TBP associated with Brf2 protein. All three protein complexes (TBP/Brf2, SNAPc, and STAF or Oct-1) bind cooperatively to the type 3 promoter, which leads to pol III recruitment and transcription initiation. 

Pol III promoters that belong to types 1 and 2 are entirely or partly located inside the transcribed DNA sequence; such promoters are called internal or intragenic [1,9]. The type 1 promoter is characteristic of only 5S rRNA genes. Such a promoter represents the sequence called internal control region (ICR); it consists of box A (+50 to +60), an intermediate element (IE, +67 to +72), and box C (+80 to +90) (the indicated box positions refer to the *Xenopus laevis* gene). ICR is recognized by TFIIIA transcription factor, and the resulting complex sequentially recruits the TFIIIC and TFIIIB factors, and finally, pol III, which transcribes the 5S rRNA gene. 

Type 2 promoters provide pol III transcription of genes coding for numerous tRNAs, vtRNA, 7SL, BC1 and BC200 RNAs, VA-I and VA-II RNAs of adenovirus, EBER1 and EBER2 RNAs of Epstein-Barr virus, and a number of other RNAs [1,10]. The promoter of this type consists of boxes A and B, 11 bp each. Box A is located around positions +12 to +22 downstream of TSS, and box B usually occupies the region from position +54 to +64, although the distance between the boxes may be slightly longer. Based on the analysis of human tRNA genes, the consensus sequence of the type 2 promoter may be presented as follows: TRGYnnARBGG (box A) and GGTTCRAnYCY (box B), where Y = C or T; R = G or A; B = T, C or G [11,12]. These boxes are similar among various eukaryotic species and among the genes of different RNA types. The large transcriptional factor IIIC (TFIIIC), consisting of five subunits recognizes boxes A and B and binds to them [9,13]. Due to protein-protein interactions this complex binds TFIIIB factor, which is formed by three subunits: TBP, Brf1, and Bdp1; as a result, TFIIIB comes close to the 5′-FS of the gene. Finally, pol III is recruited due to the interaction of the 17-subunit RNA polymerase with Brf1. 

Besides boxes A and B, the tRNA genes of plants and some yeast (*S. pombe*) have a TATA box or a very similar sequence at position −30/−24 [14]. The TATA box promotes TFIIIB recruitment, due to direct binding of TBP to the DNA. Among animals, tRNA genes with rare exceptions [15], do not have TATA boxes, and the sequences at the corresponding positions (around −30/−24) in most cases have little resemblance to TATA boxes [14,16,17]. Apart from tRNA genes, several ncRNA genes of animals and viruses also possess a type 2 promoter. All of them lack a canonical TATA box, but in the corresponding region (about −30/−24), they contain sequences resembling a TATA box. Such TATA-like boxes are not capable of binding TBP [18,19]. It was shown that changing such TATA-like boxes of 7SL RNA and EBER2 genes to more GC-rich sequences significantly reduced their expression level, which indicates an important role of these boxes for pol III transcription [20,21]. In addition to TATA-like boxes, the genes with type 2 promoters may contain recognition sites of transcription factors CREB/ATF (the genes of 7SL, EBER 1 and 2, vt RNAs, around −50 position) and Sp1 (EBER 1 and 2 RNA genes, positions −66/−60), and their deletion negatively impacts transcription [20,21,22].

The objects of this research were the murine genes of two small nuclear RNAs (4.5SH and 4.5SI) and two types of Short Interspersed Elements (SINEs), B1 and B2, which are multi-copy mobile genetic elements of rodents. These four types of DNA sequences contain A and B boxes that form type 2 pol III promoters, and are capable of transcription by this RNA polymerase [23,24,25]. The 4.5SH and 4.5SI RNAs were found and sequenced a long time ago [26,27]. Although these RNAs have similar length (94 and 98 nt, respectively), their nucleotide sequences do not resemble each other, and their genes emerged in evolution independently. The 4.5SH RNA is present in rodents of four families: Muridae (mice and rats), Cricetidae (hamsters, gerbils, and voles), Spalacidae (mole rats and bamboo rats), and Dipodidae (jerboas and birch mice) [28]. The 4.5SI RNA is characteristic of the same families of rodents, excluding Dipodidae, which indicates its later appearance in the evolution [29]. In the genomes of mice and rats, there are three 4.5SI RNA genes located 4–40 kbases one from another [30]. There are hundreds of 4.5SH RNA gene copies in the genome, and each gene is a part of a long (4–5 kbases) repeat sequence with tandem organization [28,31]. A large number of 4.5SH and 4.5SI RNA molecules can be found in various cells and organs of mouse-like rodents, and they are localized predominantly in the cellular nuclei [32]. The 4.5SI RNA is stable in the cell, whereas 4.5SH RNA is characterized by rapid turnover [33]. The 4.5SH RNA level in cells increases significantly upon heat shock exposure [34]. The functions of these RNAs remain unclear, though it was reported that 4.5SH RNA may delay the export of some mRNAs from the nucleus into the cytoplasm [35].

SINEs are intrinsic to the genomes of most multicellular organisms (see review [36]). Their length ranges from 100 to 600 bp. Due to the reverse transcription of SINE RNA performed by the enzyme encoded by Long Interspersed Elements (LINEs), SINE copies in the new sites of the genome are formed [37,38]. In mammalian genomes, the number of SINE copies may reach a million. B1 and B2 of mouse, as well as human Alu, were the first SINEs discovered and described [23,39,40]. It turned out that B1 can be found in the genomes of all rodents [41], whereas B2 is present only in the Muridae, Cricetidae, and Spalacidae families [42], which indicates that this SINE has evolved later. Most SINEs, including B2, descended from tRNA molecules. On the contrary, B1 and Alu are unique SINEs, tracing their origin to 7SL RNA [43]. Due to their origin from tRNAs or 7SL RNA, these and most other SINEs contain the type 2 pol III promoter. In the context of this study, it is noteworthy that B1 and B2 seem to serve as evolutionary predecessors of the above-mentioned 4.5SH and 4.5SI RNA genes. This is true without a doubt for 4.5SH RNA genes, which are very similar to one of the ancient B1 variants named pB1d10 [36,43]. However, this is less evident in case of 4.5SI RNA and B2: only their first 30 nucleotides are similar [42]; yet, the evolutionary link between the B2 and 4.5SI RNA genes seems quite possible, considering that they are present among the same rodents (Muridae, Cricetidae, and Spalacidae).

Apparently, the first 4.5SH RNA gene originated from one of the pB1d10 copies, and during evolution, several substitutions in the sequence of the pB1d10 copy emerged, along with some changes in the 5′-FS, which optimized the transcription and perhaps made it regulated. Similar events could also have happened in the case of the B2 and 4.5SI RNA gene, but with more substantial restructuring of the transcribed sequence itself. 

The goal of the present work is to study the contribution of 5′-FSs of murine 4.5SH and 4.5SI RNAs—as well as a number of B1 and B2 SINE copies—to the effectiveness of pol III transcription. In all cases, the importance of the −31/−24 region was shown. The impact of these regions on SINE transcription has not been reported before. The effect of substitution of each of the nucleotides from a TATA-like box on the transcription of 4.5SH and 4.5SI RNA genes was examined. The data obtained allowed us to establish the requirements for nucleotide sequences of such boxes. In 5′-FSs of 4.5SH and 4.5SI RNA genes, we found potentially active regulatory regions, similar to the recognition sites of transcription factors CREB/ATF [44], C/EBP [45], and Sp1 [46]. 

## 2. Results

### 2.1. The Deletion Analysis of 5′-Flanking Sequence of Murine 4.5SH RNA Gene

**Figure 1:**
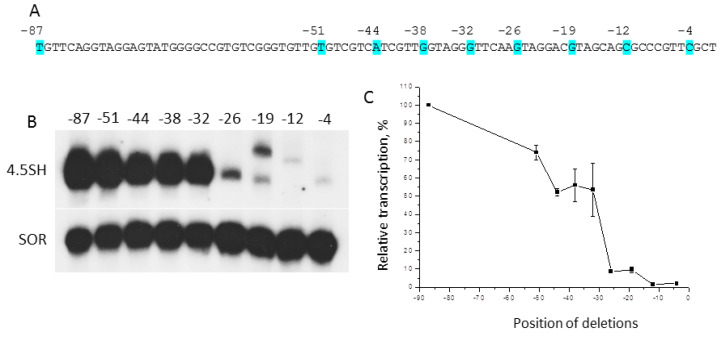
Effect of deletions in 5′-FS of 4.5SH RNA gene on its transcription in transfected HeLa cells. (**A**) 5′-FS of murine 4.5SH RNA gene. The nucleotides at the edges of deletions are marked in blue. (**B**) 4.5SH RNA gene transcripts revealed by Northern blot hybridization. The numbers above the lanes indicate the length of the remaining 5′-FS of the gene in the transfected constructs. Shrew SOR Short Interspersed Element (SINE) was used as a control for transfection. (**C**) The graph of transcription rate versus the length of the remaining 5′-FS of 4.5SH RNA gene (error bars, SD, *n* = 3).

To reveal sites in the upstream sequence of the 4.5SH RNA gene important for its transcription by RNA-polymerase III, we obtained a number of constructs where this sequence was shortened (Figure 1A). Human HeLa cells (see comments in 5.2.) were transfected by these constructs, and then gene transcription effectiveness was estimated by measuring the cellular level of 4.5SH RNA, using Northern blot hybridization. The transcription gradually decreased to 50%, while the 5′-FS was shortening from 87 bp to 32 bp; however, when it was reduced to 26 bp, the transcription dropped to 10% from the initial level (Figure 1B,C). Further shortening of 5′-FS in constructs −19 and −12 led to a change of RNA length. The results obtained indicate that: (i) −87/−32 region probably contains more than one element involved in the transcription regulation; (ii) 5′-FS of 4.5SH RNA gene between positions −32 and −26 is very important for its effective transcription; (iii) the change of native 5′-FS to the vector polylinker may shift the transcription start site. 

### 2.2. The Comparison Analysis of 5′-FSs of 4.5SH and 7SL RNA Genes

**Figure 2:**
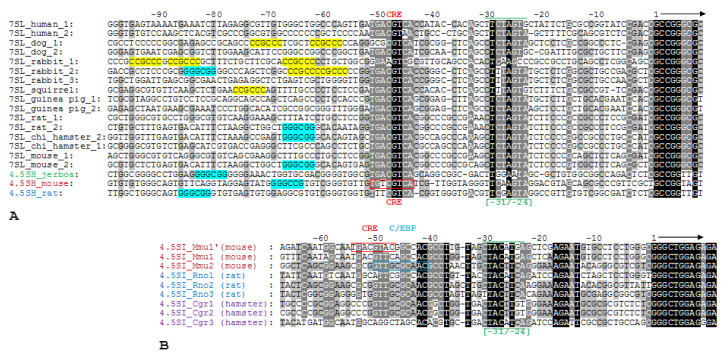
5′-FSs of 4.5SH RNA, 7SL RNA, and 4.5SI RNA genes from the genomes of different species. (**A**) 5′-FSs of 4.5SH RNA genes of rat, mouse, and jerboa are aligned with 5′-FSs of 7SL RNA genes of the mouse, Chinese hamster, rat, Guinea pig, squirrel, rabbit, dog, and human (in most cases two variants of 7SL RNA gene are showed). Two conserved regions correspond to the TATA-like box (−31/−24) and CRE (−51/−44). CRE in 5′-FS of mouse 4.5SH RNA gene is boxed. Opposite orientations of Sp1 recognition sites are highlighted in blue and yellow. (**B**) Alignment of 5′-FSs of three 4.5SI RNA gene variants from the genomes of mouse, rat, and Chinese hamster. The TATA-like box (−31/−24) is marked by lines above and below the alignment. The octamer similar to CRE in *Mmu1′* gene is boxed in red. The C/EBP recognition site in *Mmu2* gene is shown in a blue frame.

Comparison of 5′-FSs of 4.5SH RNA genes of mouse, rat, and jerboa to the corresponding sequences of 7SL RNA genes of various mammals (mouse, rat, Chinese hamster, Guinea pig, squirrel, dog, and human) revealed a significant similarity of some regions (Figure 2A). The first region with consensus CTCTAGTA for 7SL RNA genes is located between positions −31 and −24. It was reported that the replacement of four nucleotides in this region (TATA-like box) of the human 7SL RNA gene reduced its in vitro pol III transcription four-fold [21]. The presence of a similar sequence in the −31/−24 region of 4.5SH RNA genes suggests its importance for the transcription of these genes. The second region is located between positions −51 and −44 and, in the case of 7SL RNA gene, has a consensus TGACGTCA. This sequence corresponds to CREB (cAMP-response element binding protein) or ATF (activating transcription factor) recognition site. Introduction of five substitutions to the CRE (cAMP-response element) site of the human 7SL RNA gene decreased in vitro pol III transcription of the gene by 2.5 times [21]. 

The discovery of a nucleotide sequence resembling CRE at −51/−44 position of 4.5SH RNA genes opens a possibility to study its impact on the transcription of these genes. Finally, in the third region (−90/−52) of most of 7SL RNA and 4.5SH RNA genes presented in Figure 2A, we discovered sequences corresponding to the conservative part of the Sp1 transcription factor recognition site in one of the two orientations: GGGCGG or CCGCCC. Such sites had not been detected in 5′-FSs of 7SL RNA genes before, probably because the majority of the studies were performed on human genes in which these regions are disturbed by single substitutions. The same applies to 7SL RNA genes of some other mammals and to murine 4.5SH RNA gene. However, in the −90/−52 region of the 4.5SH RNA genes of rat and jerboa, there is a canonical site GGGCGG (Figure 2A) which, perhaps, is involved in the regulation of these gene’s transcription.

### 2.3. Influence of Nucleotide Substitutions in TATA-Like Box of 4.5SH RNA Gene on Its Transcription

**Figure 3:**
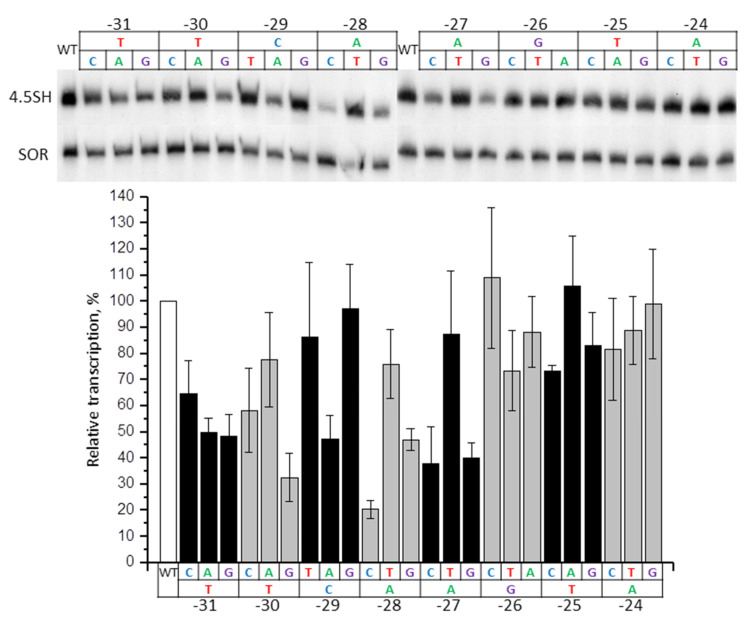
Effect of single nucleotide substitutions in −31/−24 box of 4.5SH RNA gene (*Mmu1′*) on its transcription. Upper panel: 4.5SH gene transcripts detected by Northern hybridization in HeLa cells transfected by constructs, with the replacement of each nucleotide by three other ones in the −31/−24 box. SOR SINE was transfected along with the 4.5SH RNA gene and was used as a control. Lower panel: quantitative analysis of the Northern hybridization data. The transcription level of the gene without nucleotide substitutions (WT) is taken as 100% (error bars, SD, *n* = 4).

We obtained 24 constructs of the murine 4.5SH RNA gene with substitutions of every single nucleotide to the other three in −31/−24 (TTCAAGTA) box. These constructs were transfected to HeLa cells; shrew SINE SOR was used as a control of transfection effectiveness. The intensity of the 4.5SH RNA gene transcription was estimated using Northern hybridization. The substitutions at the three positions closest to TSS (−24, −25, −26) either had a weak impact on the transcription, or did not influence it at all (Figure 3). Some substitutions of the next four nucleotides (−27A > G or C, −28A > C or G, −29C > A, and −30T > G) led to a significant (2.5–5 times) transcription decrease. Based on the localization in the 4.5SH RNA gene (−30/−27), and analogy with other genes transcribed by RNA polymerase III and possessing TATAAAA or TATA-like boxes, one may suggest that these are the nucleotides corresponding to the TATA tetramer. Finally, all the changes (−31T > C, A or G) of the eighth nucleotide, which is not included in the TATA-like box (at least formally) had a moderate negative effect (about 2 times) on gene transcription (Figure 3). Thus, the nucleotides from position −31 to −27 have a significant impact on the transcription effectiveness, whereas the contribution of other nucleotides of −31/−24 box is relatively weak. 

**Figure 4:**
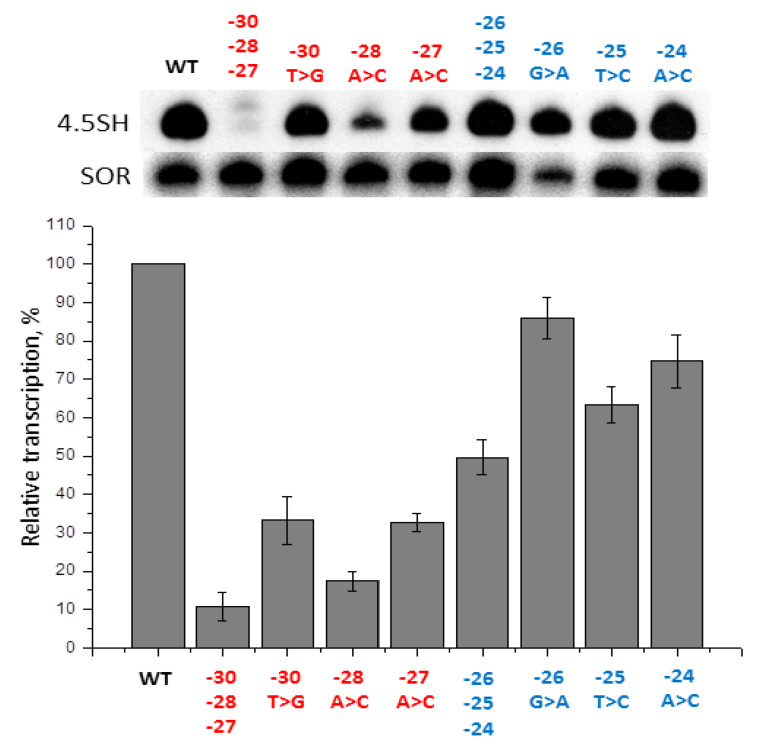
Amplification of the effect caused by nucleotide substitutions in −31/−24 box of 4.5SH RNA gene. Upper panel: 4.5SH-gene transcripts detected by Northern hybridization in cells transfected by constructs, with substitutions of one or three nucleotides in the left (−30, −28, −27) and in the right (−26, −25, −24) halves of the box. Substitutions and their positions are indicated above the lanes. Lower panel: quantitative analysis of the Northern hybridization data. The level of gene transcription without nucleotide substitutions (WT) is taken as 100% (error bars, SD, *n* = 3).

The aim of the next experiment was to determine whether the effects of different nucleotide substitutions in −31/−24 box were additive. The combination of the three mutations with weak influence on transcription (−26G > A, −25T > C, −24A > C) was introduced together in a construct of 4.5SH RNA gene, that was used to transfect HeLa cells. As expected, the effectiveness of the transcription of this construct appeared to be lower than that of each of the constructs with single nucleotide substitutions (Figure 4). The effects of the mutations added up to a large extent: three-nucleotide mutation reduced transcription by 2.0 times, which is close to the expected value, based on the effects of each of the single nucleotide substitutions (1.2 × 1.5 × 1.3 = 2.3 times). A similar experiment was performed with three mutations (−30T > G, −28A > C, −27A > C) that demonstrated strong effects. When introduced together in a 4.5SH RNA gene construct they caused a more explicit effect on transcription than each of these mutations alone (Figure 4). However, the mutation effects added up only partly: a three-nucleotide mutation reduced the transcription by 10 times, which is significantly less than a 50-fold reduction expected in a case when the effects of three one-nucleotide mutations are added up fully (3.0 × 5.5 × 3.0). Such a non-additive outcome of combined mutation effects indicates that the basic level of transcription may be sustained by various nucleotide sequences at the −31/−24 position. However, for the most effective transcription, the requirements for the sequence at this position are rather stringent. 

### 2.4. Influence of Nucleotide Substitutions in TATA-Like Box of 4.5SI RNA Gene on its Transcription

**Figure 5:**
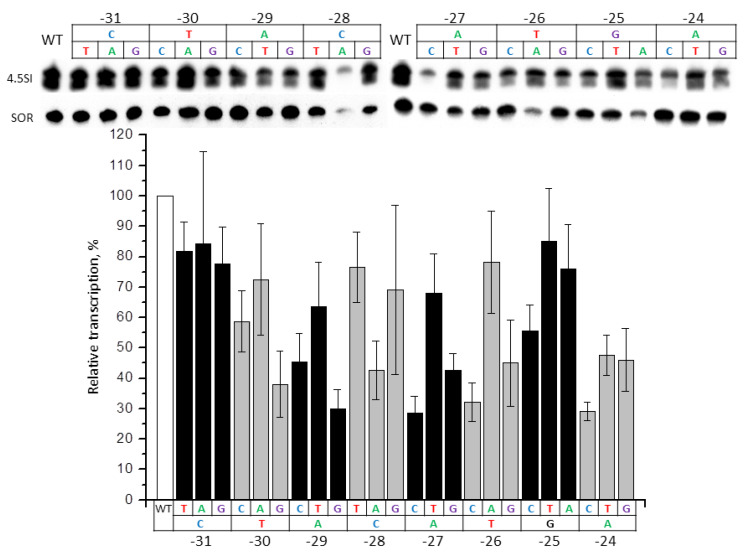
Effect of single nucleotide substitutions in box −31/−24 of the murine 4.5SI RNA gene on its transcription. Upper panel: 4.5SI-gene transcripts detected by Northern hybridization in cells transfected by constructs with the replacement of each nucleotide by three other ones in the −31/−24 box. SOR SINE was transfected along with the 4.5SI RNA gene, and used as a control. Lower panel: quantitative analysis of the Northern hybridization data. The level of gene transcription without nucleotide substitutions (WT) is taken as 100% (error bars, SD, *n* = 4).

We have previously discovered that replacement of −31/−24 region of murine 4.5SI RNA gene with different sequences from pGEM-T vector polylinker may alternatively reduce the gene transcription substantially, or not influence it negatively at all [47]. It turned out that, to a large extent, this depends on GC-content of the replacing sequence. To evaluate the contribution of each of the nucleotides in the −31/−24 region of murine 4.5SI RNA gene (*Mmu 1′*) to the transcription effectiveness, 24 constructs of this gene with single nucleotide substitutions in the −31/−24 box (CTACATGA) were obtained. The RNA isolated from HeLa cells transfected with these constructs was analyzed by Northern hybridization (Figure 5). Substitutions of the nucleotide at position −31 had a weak influence on the 4.5SI RNA gene transcription, or did not influence it at all. The same partly applies to position −25, though −25G > C substitution reliably lowered the transcription by 1.8 times. At least one of the changes in the rest of the positions reduced the gene transcription more significantly (by 2.3–3.5 times). Such substitutions are: −30T > G, −29A > G or C, −28C > A, −27A > C or G, −26T > C or G, and −24A > C or G or T. Thus, nucleotides in all these positions are important for the effective transcription of the 4.5SI RNA gene. Box −31/−24 consensus of nine 4.5SH RNA genes of mouse, rat, and Chinese hamster (three for each species) (Figure 2B) may be presented as follows: (C/t)TAC(T/A)T(C/G)(A/t), where alternative nucleotides in each position are indicated in brackets via slash, lowercase letters are the minor variants of nucleotides, which are present in only one or two of the nine genes. Overall, the results of our experiments with single nucleotide substitutions do not contradict the structure of this consensus. At the same time, box −31/−24 of 4.5SI genes looks rather conservative, and many substitutions that do not practically influence the transcription effectiveness (for example, −30T > A, −29A > T, −28C > T or G, −26T > A) do not occur in this box. Probably the evolutionary selection of this box went not only in the direction of increased transcription level, but also in some other directions, for example, the ability for regulation or specificity of action.

**Figure 6:**
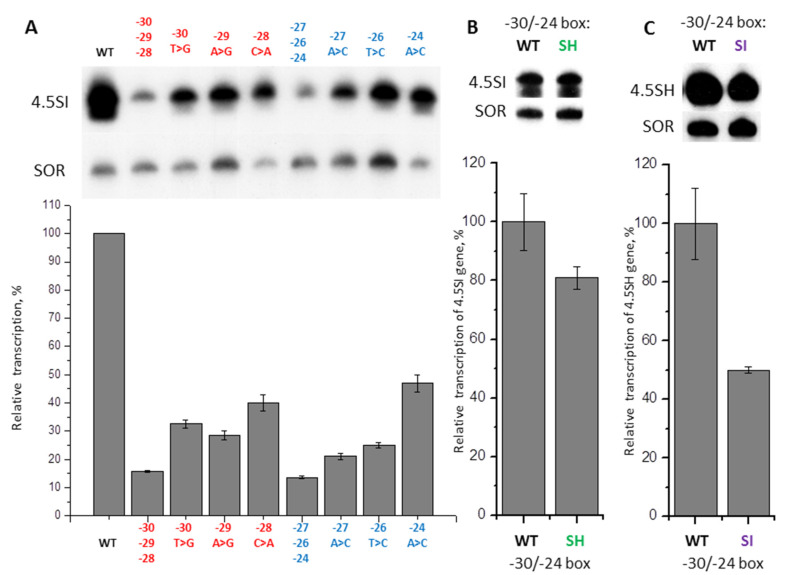
(**A**) Amplification of the effect caused by substitution of nucleotides in box −31/−24 of 4.5SI RNA gene. Upper panel: transcripts of 4.5SI gene detected by Northern hybridization in cells transfected by constructs with substitutions of one or three nucleotides in the left (−30, −29, −28) and in the right (−27, −26, −24) halves of the box. The substitutions and their positions are indicated above the lanes. Lower panel: quantitative analysis of Northern hybridization data. The transcription level of the gene without nucleotide substitutions (WT) is taken as 100% (error bars, SD, *n* = 3). (**B**) The effect of the −30/−24 sequence replacement in 4.5SI RNA gene by the −30/−24 box from the 4.5SH RNA gene on its transcription. (**C**) The effect of replacement of the −30/−24 sequence in 4.5SH RNA gene by the −30/−24 box from 4.5SI RNA gene on its transcription.

We examined whether the effect of nucleotide substitutions in the −31/−24 box on the 4.5SI RNA gene transcription added up (Figure 6A). Simultaneous replacement of three nucleotides (−30T > G, −29A > G, −28C > A) in the left part of this box reduced transcription by 6.4 times, which is less than could be expected, based on the effects of each of these three mutations alone (3.1 × 3.5 × 2.5 = 27 times). The similar result was obtained for substitutions of three nucleotides (−27A > C, −26T > C, −24A > C) in the right part of box −31/−24: the three-nucleotide substitution decreased the transcription by 7.4 times, while single nucleotide mutations reduced it by 4.8, 4.0, and 2.1 times, respectively, which should have led to 40-fold reduction in case of fully additive effect. Similar to the 4.5SH RNA gene, these results may be interpreted as follows. Box −31/−24 of 4.5SI RNA gene is optimized during evolution for effective transcription, and that is why single nucleotide substitutions substantially reduce it; however, subsequent introduction of mutations has an insignificant additive effect as, apparently, the requirements of the nucleotide sequence of the box to render its moderately-effective transcription are quite low. 

Finally, we performed experiments to study the influence of mutual exchange of the −31/−24 boxes between the 4.5SI and 4.5SH RNA genes on their transcription. The 4.5SI RNA gene containing the −31/−24 box of the 4.5SH RNA gene was transcribed at a 20% lower level than the wild type gene (Figure 6B). The 4.5SH RNA gene with the box −31/−24 from the 4.5SI RNA gene was transcribed twice weaker than the original gene (Figure 6C). Apparently, nucleotide sequences of −31/−24 boxes were optimized during evolution to function with their native genes. 

### 2.5. Contribution of −31/−24 Region to the Efficiency of SINE B1 Transcription

It is well known that new SINE copies arise in various regions of the genome, due to the reverse transcription of their pol III transcripts performed by the enzyme encoded in LINE. The sequences flanking SINE copies are very diverse and have little in common. However, it should be noted that the 10–15 bp long genomic sequences adjacent to mammal SINEs may be substantially enriched by A residues. However, completely different (random) nucleotide sequences appear at the −31/−24 position upstream SINE copies. Therefore, we wanted to assess to what extent the regions located at this position are important for SINE transcription provided that −31/−24 boxes significantly contribute to the effective transcription of the 4.5SH and 4.5SI RNA genes. 

**Figure 7:**
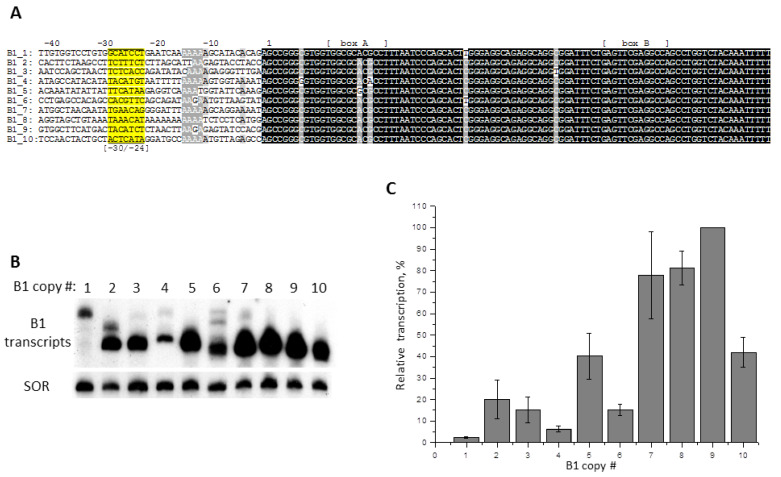
Transcription of 10 similar copies of B1 SINE from murine genome introduced to HeLa cells. (**A**) Nucleotide sequences of the 3′-truncated B1 copies (mini-B1s) with their 5′-FSs. The A and B boxes of pol III promoter and the −30/−24 region sequences are labeled. (**B**) The transcripts of mini-B1 detected by Northern hybridization in the transfected cells. The numbers of B1 copies are indicated above the lanes. (**C**) Quantitative analysis of Northern hybridization results. The level of B1_9 copy transcription is taken as 100% (error bars, SD, *n* = 3).

In the mouse genome, ten SINE B1 copies with similar nucleotide sequences but different flanking sequences were found by means of bioinformatics methods. They were amplified using primers to the flanking sequences and cloned. Then using PCR each B1 copy was shortened, and the remaining 5′-end part of the SINE containing A and B boxes of pol III promoter was furnished with pol III transcription terminator (TTTTT), and cloned again in pGEM-T plasmid. It is much more convenient to work with such mini-B1 (Figure 7A) than with the full-size B1, whose transcripts appear as a smear, rather than a band, on denaturing electrophoresis gel, which, apparently, is due to the incomplete disruption of its secondary structure [48]. Each of the ten cloned mini-B1 was transfected into cells, and the transcription of these SINEs was analyzed by Northern hybridization (Figure 7B,C). The efficiency of the B1 copies transcription was very different: the transcription of B1_5, B1_7, B1_8, B1_9, and B1_10 was most intense, that of B1_2, B1_3, and B1_6—moderate, whereas B1_1 and B1_4—very weak. It should be noted that the transcripts of the two latter B1 copies were longer than expected. Such extension of these transcripts cannot be explained by additional sequence on 3′-end, as mini-B1 in all constructs were equipped with the same strong terminators of pol III transcription. The RNA elongation is likely caused by transcription of B1_1 and B1_4, starting from the sites located upstream from the standard TSS. In fact, we had previously reported [49] on the experimentally confirmed examples of such transcription impairment, in the case of complete replacement of 5′-flanking sequence of 4.5SH RNA gene. 

**Figure 8:**
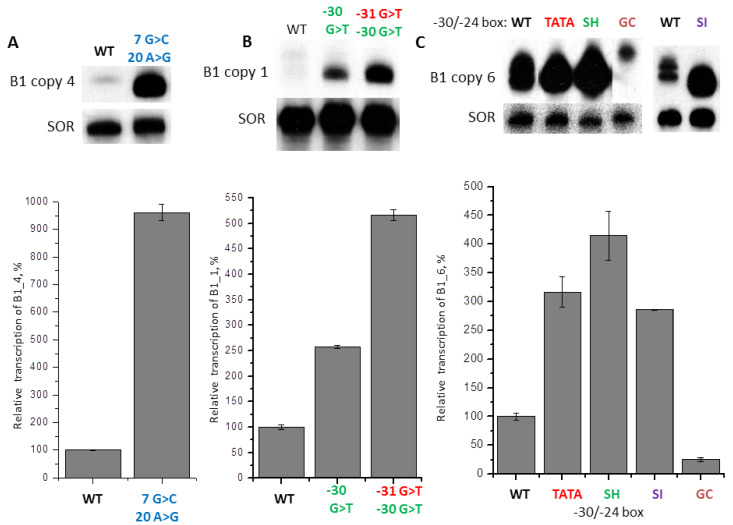
(**A**) B1_4 transcription increases after two substitutions at the beginning of SINE, including A to G replacement in box A of pol III promoter (error bars, SD, *n* = 3). (**B**) B1_1 transcription increases after G to T replacements at positions −30 and −31. The transcription level of B1 with the original 5′-FS (WT) is taken as 100% (error bars, SD, *n* = 3). (**C**) B1_6 transcription is modulated by replacement of −30/−24 sequence by TATA box, −30/−24 boxes of genes 4.5SH RNA and 4.5SI RNA, and GC-rich heptamer. The transcription level of B1_6 with the intact 5′-FS (WT) is taken as 100% (error bars, SD, *n* = 3).

One may suggest that the low level of B1 transcription and, in some cases, TSS shift, may be caused by the sequences located at −31/−24 position that are not quite suitable for functioning as TATA-like boxes. However, impairment in the internal promoter (A and B boxes) of pol III may also be the reason for that. It should be noted that in box A of a weakly transcribed B1_4 copy there is A instead of G at position 20, which can, apparently, impair the function of this box. We substituted two nucleotides (7G > C and 20A > G) in B1_4 to make the sequence of this copy identical to the consensus of mini-B1 (Figure 8A). Upon transfection, the construct with these substitutions demonstrated a 10-fold increase of transcription level, as compared to the original B1_4 construct; additionally, the RNA length matched more accurately the length of the standard transcript. Thus, the low ability for transcription of B1_4 copy is determined by mutations within the SINE itself, and the main role in it is likely played by the nucleotide substitution in box A. It should be noted that copy B1_5 also has a mutation in box A: a conserved nucleotide A at position 18 is replaced with G (Figure 7A). Nevertheless, B1_5 was actively transcribed, and further transcription enhancement can be expected after a reverse G > A substitution. Apparently, the 5′-FS does not interfere with efficient pol III transcription of this B1 copy. 

The poorly transcribed SINE copy B1_1 (Figure 7A) has a single nucleotide substitution (38C > T). It is located between boxes A and B, and should not have any influence on the effectiveness of SINE transcription. That is why we focused on the −31/−24 region. Nucleotide sequences of this region in different B1 copies have little resemblance, but all of them allowed effective or moderate transcription of B1. The only exception is, apparently, the B1_1 copy, which has G residues uniquely located at positions −30 and −31 (Figure 7A). It should be noted that, in various 7SL and 4.5SH RNA genes, only pyrimidines were present at these positions (Figure 2A). We obtained two constructs of B1_1: in one of them −30G was replaced by T, and in the other construct, both Gs (−30 and −31) were replaced by T residues. In transfection experiments these constructs showed a 2.5- and 5-fold increased transcription, respectively, as compared to the original B1_1. Additionally, judging by the length of RNA, B1_1 started to transcribe from the standard TSS after introduction of these substitutions. Thus, this example demonstrates an important role of nucleotides at positions −30 and −31 for the effective transcription of B1 SINE. 

Another series of experiments was performed with the B1_6 copy, which had a moderate ability for pol III transcription (Figure 7B,C). We obtained constructs with the −30/−24 region (CACGTTC) replaced by TATA-box (TATAAAA), GC-rich sequence (GGCGGCC) or the corresponding regions of the 4.5SH RNA gene (TCAAGTA, called SH-box, see below) and the 4.5SI RNA gene (TACATGA, SI-box below). A Northern-blot analysis of RNA from transfected HeLa cells showed that TATA box, SH-box, and SI-box increase B1_6 transcription by 3.2, 4.2, and 2.7 times, respectively (Figure 8C). On the contrary, GC-rich sequence reduced the transcription 5-fold, with the resultant RNA being slightly longer than the original B1_6 transcript (Figure 8C), indicating that GC-rich sequence leads to TSS upstream shift. Thus, the data obtained demonstrate the significance of −30/−24 region for pol III-transcription of SINE B1. 

### 2.6. Contribution of −31/−24 Region to the Efficiency of SINE B2 Transcription

**Figure 9:**
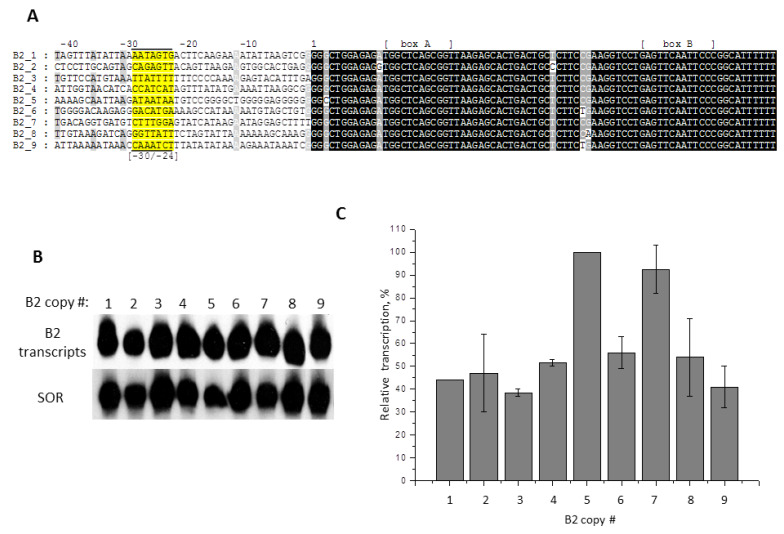
Transcription of nine similar B2 SINE copies from murine genome introduced into HeLa cells. (**A**) Nucleotide sequences of the 3′-truncated B2 copies (mini-B2) with their 5′-FSs. Boxes A and B of pol III promoter and −30/−24 sequences are labeled. (**B**) Mini-B2 transcripts detected by Northern hybridization in the transfected cells. The numbers of B2 copies are indicated above the lanes. (**C**) Quantitative analysis of Northern hybridization data. B2_5 transcription level is taken as 100% (error bars, SD, *n* = 3).

We studied another murine SINE, B2, applying the approach similar to that which we had used for the −31/−24 region of the B1 copies. By computer search, we found nine copies of B2 with very similar nucleotide sequences, but different FSs. These copies, together with the adjacent sequences, were amplified by PCR; in the second round of PCR, 5′-FSs were amplified together with the head part of B2 and a transcription terminator at the end (Figure 9A). PCR-products were cloned in pGEM-T, the obtained plasmids with mini-B2 were transfected into HeLa cells and then transcription of these SINEs was analyzed by means of cellular RNA hybridization with B2-specific probe (Figure 9B). All nine B2 copies were transcribed intensely and their transcription efficiency differed no more than 2.5-fold (Figure 9C). The sequences of −31/−24 region of these copies varied (Figure 9A). In three of them (B2_1, B2_3, and B2_5), these 8-nucleotide sequences resembled TATA boxes and contained 8, 7 or 6 A/T-nucleotides. In the rest of the copies, the −31/−24 regions contained less A/T-nucleotides—four or five. In two cases (B2_6 and B2_8), there were G residues at positions −31 and −30, but that did not interfere with the effective B2 transcription. As was shown above for B1_1, the presence of G in positions −31 and −30 strongly reduced the effectiveness of transcription and shifted TSS (Figure 8B). Apparently, the requirements for the nucleotide sequences of −31/−24 region are less strict for B2 SINE than for B1 SINE. 

**Figure 10:**
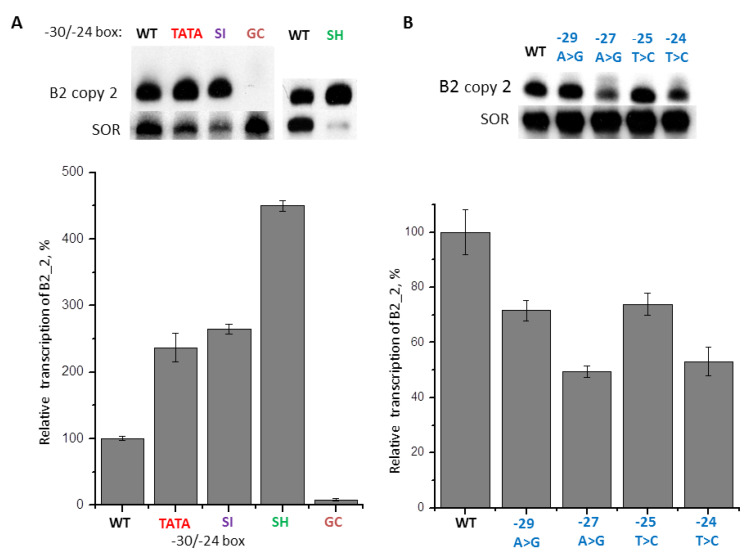
(**A**) B2_2 transcription is modulated by replacement of the −30/−24 sequence by TATA box, boxes −31/−24 of 4.5SI RNA and 4.5SH RNA genes, and GC-rich heptamer. The transcription level of B2_2 with the intact 5′-FS (WT) is taken as 100% (error bars, SD, *n* = 3). (**B**) Effect of single-nucleotide substitutions in the −30/−24 region of B2_2 copy on its transcription. Substitutions and their positions are indicated above the lanes and on the diagram (error bars, SD, *n* = 3).

For further research, we have chosen a B2_2 copy with a moderate level of transcription (about 50% from B2_5 transcription). Constructs with the −30/−24 region (CAGAGTT) replaced by TATA box (TATAAAA), GC-rich sequence (GGCGGCC), or the corresponding regions of 4.5SI RNA gene (SI-box, TACATGA) and 4.5SH RNA gene (SH-box, TCAAGTA) were obtained. Transfection of HeLa cells with subsequent Northern-blot analysis of RNA showed that TATA box, SI-box, and SH-box increased B2_2 transcription by 2.3, 2.6, and 4.4 times, respectively (Figure 10A). On the contrary, the GC-rich sequence almost completely suppressed the transcription. Furthermore, we checked the influence of A to G and T to C substitutions in the −30/−24 region on B2_2 transcription. All four such mutations reliably lowered transcription: −29A > G and −25T > C—down to 70% from the control, −27A > G and −24T > C—down to 50% from the control (Figure 10B). The cumulative impact of these four substitutions should suppress transcription to about 12% (0.7×0.7×0.5×0.5×100%) from the control, which is consistent with the strong influence of replacement of the whole −30/−24 region to the GC-rich sequence (Figure 10A). Thus, the general tendency revealed here is that strong increase of the GC composition in the −30/−24 region lowers B2 transcription, whereas, in the case of moderate GC composition in this region, B2 transcription may be very active. In fact, SI and SH boxes (G + C=25%) inserted at the −30/−24 position of B2 induced pol III transcription even stronger than the canonical TATA box. Most likely, during evolution, the −31/−24 regions in the 4.5SI and 4.5SH genes were optimized, whereas among B2 copies, these sequences have a random origin and, apparently, are not usually subjected to selection. 

### 2.7. Additional Regulatory Elements in 5′- Flanking Sequences of 4.5SH Genes

**Figure 11:**
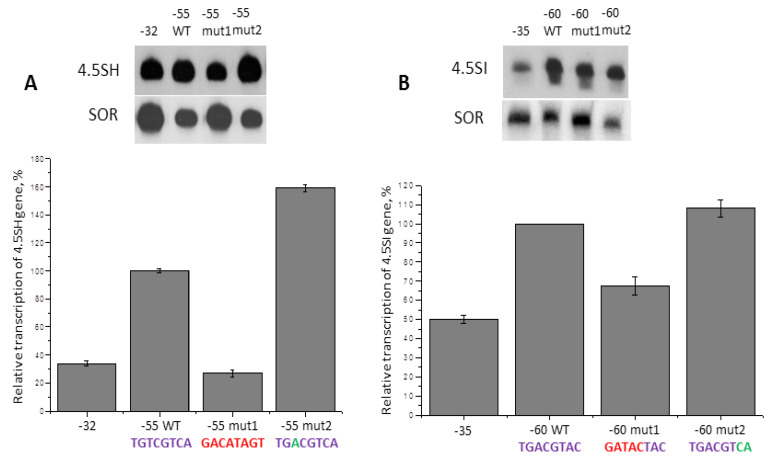
Effect of deletions and substitutions of cAMP-response element (CRE)-like sites in murine 4.5SH RNA (**A**) and 4.5SI RNA (**B**) genes on their transcription. The constructs with deletions up to positions −32 or −35 lack CRE sites, while the constructs −55WT or −60WT contain them. These sites in mut1 are inactivated by nucleotide substitutions (red), while in mut2 they are converted to canonical CRE sites by replacing one or two nucleotides (green). Error bars, SD, *n* = 3.

As it was noted above (Chapter 2.2) we found a sequence resembling cAMP-response element (CRE) at the −51/−44 position of the 4.5SH RNA genes (mice, rats, and jerboas). This site in the murine gene (TGTCGTCA, in frame on Figure 2A) differed by one nucleotide from the canonical CRE (TGACGTCA). To check the ability of this sequence to influence the pol III transcription of the 4.5SH RNA gene we obtained: (i) a construct containing the 4.5SH RNA gene of mouse at 5′-FS 55 bp long; (ii) the same construct, but with a −49T > A substitution, which should ‘improve’ CRE site; (iii) a construct with replacement of the whole −51/−44 region to a dissimilar sequence (GACATAGT); (iv) the 4.5SH RNA gene construct where 5′-FS ends at position −32. Experiments on transfection of HeLa cells with these constructs showed that the deletion of the −55/−32 region or replacement of the whole −51/−44 sequence reduces 4.5SH RNA gene transcription about 3-fold, whereas −49T > A substitution increases the transcription by 1.6 times (Figure 11A). Thus, CRE site contributes significantly to the efficiency of the 4.5SH RNA gene transcription, and the substitution optimizing the nucleotide sequence of CRE additionally ameliorates it. It is worth noting that the 4.5SH RNA gene of jerboa contains just such a perfect CRE site, whereas the rat gene contains additional mutation −50G > T in this site (Figure 2A). 

By analyzing nucleotide sequences (see Chapter 2.2, Figure 2A), we discovered in the −90/−52 region of most 7SL RNA and 4.5SH RNA genes sites corresponding to the GC-box consensus (GGGCGG or CCGCCC), which can potentially bind pol II transcription factors, such as Sp1, Krox/Egr, Wilms’ tumor, MIG1, and CREA [50]. In the 4.5SH RNA genes of rat and jerboa, there is one GGGCGG site, and in the murine gene this site differs by one nucleotide—GGGCCG. To evaluate the contribution of the GC-boxes to the 4.5SH RNA genes transcription, we obtained the following constructs based on these genes of mouse, rat, and jerboa: (i) gene constructs with 5′-FSs 87 bp long; (ii) analogous constructs, but with the GC-box replaced by another sequence—AAATAA; (iii) constructs of genes with 5′-FSs 51 bp long. This third type of construct was obtained in order to delete the whole −87/−51 region containing, besides the GC-box, other GC-sequences, which, apparently, may also function as GC-boxes. Transfecting HeLa cells by three types of constructs with the genes of three rodent species, we obtained the following results (Figure S2). The deletion of the −87/−51 region reduced transcription but not strongly—by 15–20%, and this reduction was statistically significant only in case of jerboa gene. After replacement of the GC-box with the AAATAA hexamer, a noticeable decrease of jerboa gene transcription by 28% occurred, however, the decrease was non-significant or was absent in case of genes of mouse and rat. Unfortunately, the obtained results do not allow us to make a clear conclusion about contribution of GC-box or the whole −87/−51 region to the transcription. Apparently, these sequences contribute little to the transcription efficiency, as compared to the input of CRE and TATA-like box, which complicates reliable evaluation of the −87/−51 region’s influence.

### 2.8. Additional Regulatory Elements in 5′-Flanking Sequences of 4.5SI RNA Genes

At position −54/−47 of two murine 4.5SI RNA genes, we found (Figure 2B) TGACGTAC (*Mmu1′*) and TGACGTTC (*Mmu1*) sequences resembling CRE: TGACGTCA (identical nucleotides are underlined). It turned out that replacement of first five nucleotides of this sequence of *Mmu1′* gene reduced its transcription by 30%, and substitution of two last nucleotides (AC > CA), making this site identical to the canonical CRE, only slightly increased the gene transcription (Figure 11B). Thus, the sequence at the −54/−47 position somewhat contributes to the increase of the 4.5SI RNA *Mmu1′* gene transcription by pol III; probably the same is correct for gene *Mmu1*. 

In the similar position (−51/−42) of the murine gene *Mmu2*, we found the GTTGCGCAAC sequence (Figure 2B), corresponding to the canonical recognition site of transcription factor C/EBP: RTTGCGYAAY [51]. The same sequence at the same position is present in two 4.5SI RNA genes (Rno2 and Rno3) of rat and in two genes (Cgr1 and Cgr2) of Chinese hamster (Figure 2B). Evolutionary conservation of this site indicates that it is significant for pol III transcription of 4.5SI RNA genes. To check this, we obtained constructs of *Mmu2* gene with the deletion or replacement of nucleotides in −51/−42 box. It turned out that the elimination of this sequence substantially (by 50%) reduces transcription of 4.5SI RNA gene (Figure S3). Perhaps, this region of 5′-FS is involved in regulation of 4.5SI RNA genes, and its influence on transcription becomes more obvious in some specific conditions. It is worth noting that C/EBP recognition sites had never been found in 5′-FS of genes transcribed by pol III. However, a remarkable example of the human miRNA gene transcribed by pol III has been recently discovered, in which box B of the internal promoter overlapped with the C/EBP recognition site, which contributed greatly to the efficient transcription of the gene [52]. 

## 3. Discussion

A TATA-binding protein (TBP) is required to initiate transcription of each of the three types of pol III promoters. TBP is a part of transcription factor TFIIIB which also includes the proteins Bdp1 and Brf1 (or Brf2 in the case of type 3 promoters). TFIIIB has two functions: (1) the unwinding of DNA in its binding region, and (2) the recruitment of pol III. TBP can bind independently to the canonical TATA box, and then successively recruit Brf1 (or Brf2) and Bdp1. However, genes transcribed by pol III from type 2 promoters in mammals and, apparently, in other animals, do not have such TATA boxes in the corresponding position (approximately −30/−25) of their 5′-FSs (see Introduction). It is known that TBP is not able to bind independently even to slightly modified TATA boxes (for example, GATAAA). Both TFIIIC and preliminary association of TBP with Brf1 are required for binding of TBP to promoter without canonical TATA box [18]. Although it had been previously clear that the sequences in the region −30/−24 of genes that do not have TATA boxes were very diverse, the requirements for such nucleotide sequences remained poorly understood. 

### 3.1. TATA-Like Boxes in 4.5SH RNA and 4.5SI RNA Genes

**Figure 12:**
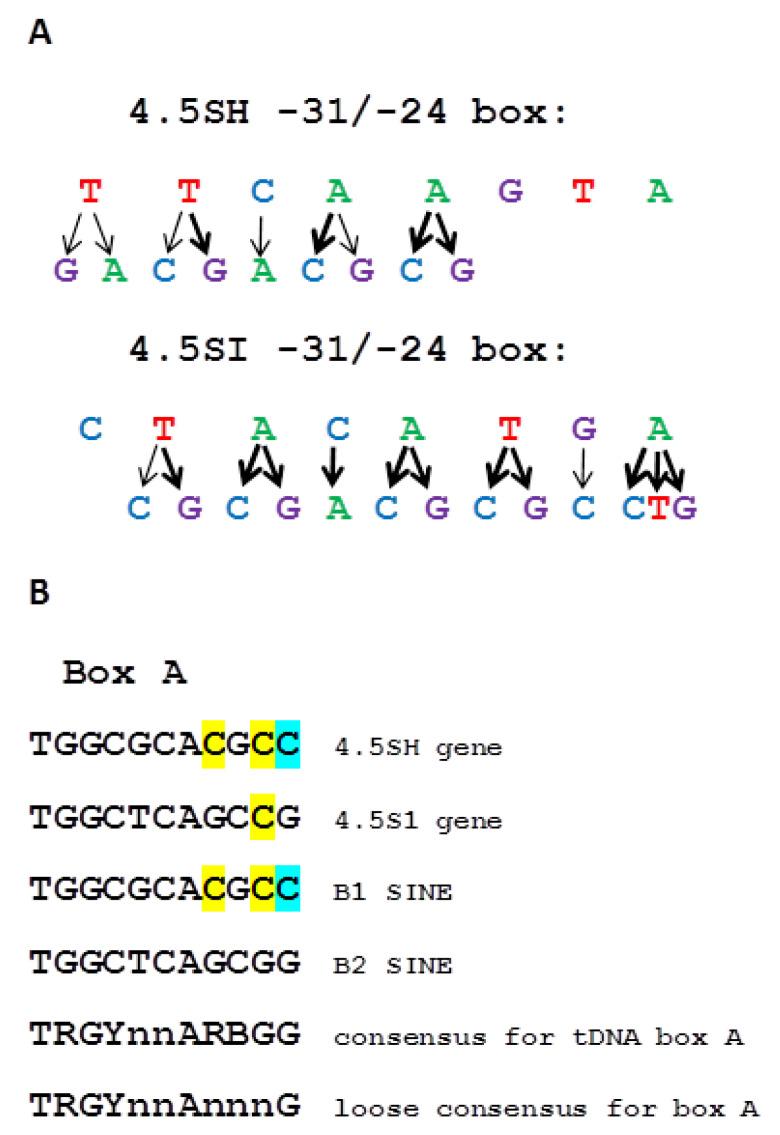
(**A**) Nucleotide sequences of −31/−24 boxes of murine 4.5SH RNA and 4.5SI RNA (*Mmu1′*) genes. The nucleotide substitutions leading to strong or moderate reduction of pol III transcription of these genes are indicated by thick and thin arrows, respectively. (**B**) Comparison of box A nucleotide sequences from murine 4.5SH RNA and 4.5SI RNA (*Mmu1′*) genes, as well as B1 and B2 SINEs with box A consensus sequences from tRNA genes and SINEs. The nucleotides differing from those in box A consensus sequence of tRNA genes are highlighted [12,53]. The C residue, which does not correspond even to the loose consensus sequence of box A, is marked in blue.

Here we found a certain sequence similarity at position −31/−24 of the 4.5SH RNA genes of mouse, rat, and jerboa (TTCGAGTA consensus) with the corresponding region of 7SL RNA genes of various mammals (CTCTAGTA consensus). It has been reported that in vitro transcription efficacy of the human 7SL RNA gene decreased after the replacement of this region, which, despite very little similarity to TATAAAA, is still called a TATA-like box [21]. We showed that the removal of a similar region in the mouse 4.5SH RNA gene led to a sharp decrease in its transcription efficacy in transfection experiments, which indicates an important role of the TATA-like box in the transcription initiation of this gene. By replacing each nucleotide with any of the other three in the TATA-like box of the mouse 4.5SH RNA gene (−31/−24: TTCAAGTA), we found that five distal nucleotides (−31/−27) are the most important. The greatest transcriptional suppression effect was caused by the replacement of T or A with C or G, however, the substitutions −31T> A and −29C> A also had a significant effect (Figure 3 and Figure 12A). As expected, the general trend is that the box −31/−24 should be AT-rich, but with conserved C at position −29.

Previously, by testing the transcription of the mouse 4.5SI RNA gene with a stepwise shortening 5′-FS, we revealed the importance of the site located at approximately −30/−24 [47]. We noticed significant differences between the graphs of change of the 4.5SI RNA and 4.5SH RNA genes’ transcription efficacy when their 5′-FSs were shortened (Figure S4). When the 5′-FS was shortened to position −19 (i.e., box −30/−24 is already absent), the 4.5SH RNA gene ceased to be transcribed, whereas the transcription of the 4.5SI RNA gene was still efficient. Our interpretation of these data is that, apparently, the internal promoter (boxes A and B) of the 4.5SH RNA gene is weaker than that of the 4.5SI RNA gene (see below), therefore, transcription of the former gene is more dependent on TATA-like box than that of the latter one. In other words, the 4.5SI RNA gene appears to be less demanding on the nucleotide sequence located at position −30/−24. In constructs with a 19-nt 5′-FS, the region (CACTAGT) of the pGEM-T polylinker occurred at the position of the TATA-like box, which, judging by the results (Figure S4), supports transcription of the 4.5SI RNA gene, but not the 4.5SH RNA gene. Nevertheless, the 4.5SI RNA gene transcription stopped almost completely when the 5′-FS was shortened to 12 nt and the GC-rich region of the polylinker appeared at position −30/−24.

Point replacement of each of the nucleotides in the TATA-like box of the mouse 4.5SI RNA gene (−31/−24: CTACATGA) to three others showed that all but the distal nucleotide (−31) contribute to transcription efficacy. In general, replacing T or A with C or G gave the greatest effect, although replacing C with A at position −28 also significantly reduced the transcription efficacy (Figure 5 and Figure 12A). It should be noted that this C residue is part of the conserved trinucleotide (TAC) in the TATA-like box of the mouse, rat, and hamster 4.5SI RNA genes (Figure 2). Probably, this trinucleotide determines the specificity of interaction of TATA-like boxes in the 4.5SI RNA genes with TFIIIB.

Our results show that the nucleotides that contribute most to the TATA-like box function have different positions in the sequences of 4.5SH RNA and 4.5SI RNA genes. In case of 4.5SH RNA, the distal section of the box is the most important (nucleotides at positions −31, −30, −29, −28, and −27), whereas in 4.5SI RNA genes, the important nucleotides are distributed more evenly over the TATA-like box (only replacements at position −31 have quite weak effect) (Figure 12A). Presumably, the observed difference in the distribution of the most effective nucleotide substitutions is somehow related to the atypical structure of box A of the internal pol III promoter of the 4.5SH RNA gene (Figure 12B). Box A of this gene has three nucleotides that distinguish it from the box A consensus of tRNA genes and SINEs, and one of these nucleotides (the last C, highlighted in blue in Figure 12B) does not meet even the least stringent requirements for boxes A [54]. Only 7SL RNA genes, SINEs B1 and Alu possess the same atypical boxes A, which is quite expected, since these SINEs and 4.5SH RNA genes originate from 7SL RNA. Apparently, this replacement of the last G by C in box A reduces its effectiveness greatly: the internal promoter of 4.5SH RNA gene is six times less effective than the promoter of the 4.5SI RNA gene (unpublished data). Since the recruitment of TFIIIB requires the preliminary association of TFIIIC with boxes A and B, the deterioration of box A could influence indirectly the interaction of TFIIIB with TATA-like box. This, probably, explains the fact that only the distal nucleotides of −31/−24 region of the 4.5SH RNA gene strongly affect the efficacy of transcription. Presumably, this also determines the sensitivity of 4.5SH RNA gene transcription (but not 4.5SI RNA gene transcription) to the replacement of the native TATA-like box with the vector polylinker (Figure 1 and Figure S4), as discussed above.

### 3.2. The Role of −31/−24 Sequence in B1 and B2 SINE Transcription

SINEs can integrate into different random genome sites, therefore their 5′-FSs are very diverse. It is believed that SINE transcription by pol III is completely dependent on boxes A and B and, in general, is independent of 5′-FSs. However, it has been reported that one actively propagating copy of SINE Alu (source gene) at position −33/−27 has a binding site of transcription factor Ap1, which in vitro activated pol III transcription of this Alu copy [55]. In another study [56], it was found that the removal of 5′-FSs up to position −12 had practically no effect on Alu transcription in vitro (three copies), reduced it moderately (two–three times, three copies) or strongly (four–five times, three copies). These data indicate that the 5′-FSs of at least some Alu copies positively affect their pol III transcription. However, another explanation of these results cannot be ruled out: the polylinker of the vector adversely affects SINE transcription in one of the two orientations of a cloned Alu copy.

Here we cloned 10 very similar copies of the mouse B1 SINE with completely different 5′-FSs. Transfection experiments showed that these copies of B1 are transcribed with different efficiency: differences could reach 50 times (Figure 7). This result clearly indicates that 5′-FSs strongly influence pol III transcription of B1. Since murine 5′-FSs in the studied constructs were rather short (65–90 bp), we suggested that the main influence on transcription should be exerted by sites at position −31/−24, which probably function as TATA-like boxes. A confirmation for this was the experiment with the B1_1 copy that was transcribed very poorly. Replacing G with T at positions −31 and −30 increased the efficacy of transcription by five times (Figure 8B). The importance of box −31/−24 for transcription was also confirmed by replacement of the −30 to −24 region in a moderately transcribed copy of B1_6 by other heptanucleotides (Figure 8C). Interestingly, the greatest increase in transcription (four times) was achieved when SH-box (TATA-like box of the 4.5SH RNA gene) was inserted into the construct. Significant increase (about three times) also had replacements by the classic TATA or SI-boxes. At the same time, substitution with a GC-rich sequence reduced transcription 5-fold. Thus, the “random” sequence at position −30/−24 of B1_6 copy is capable of supporting transcription which, in this case, is noticeably weaker than TATA-like boxes of pol III-transcribed genes, but stronger than sequences containing only G and C. Apparently, this can apply to most of the copies of B1 with normal A and B boxes of pol III promoter.

Similar experiments were conducted with B2 SINE. In this case, all nine SINE copies studied were transcribed very intensively, and the differences between the copies were no more than 2.5 times (Figure 9). An interesting example is B2_5: the region from −20 to −1 nucleotide in this copy is very GC-rich; it consists mainly of G residues and contains only one T and one A. Yet, B2_5 is transcribed intensively, apparently due to the AT-rich sequence similar to typical TATA box located at position −30/−24. To obtain additional evidence for significant contribution of the −30/−24 region to transcription efficiency, it was replaced with other sequences in a moderately transcribed B2_2 copy. Substitution with a GC-rich sequence suppressed the transcription almost completely, whereas substitution with TATA, SI-, and, especially, SH-box increased the transcription efficacy greatly (Figure 10A). Thus, specific −30/−24-boxes of pol III-transcribed genes can act much more efficiently than the “random” 5′-flanking SINEs sequences. SH-box of 4.5SH RNA gene is especially effective, probably because it must compensate for the “weakness” of the internal pol III promoter, prompted by a significant deviation of box A structure from the consensus (Figure 12B).

### 3.3. Changed −31/−24 Box may Shift the TSS

In experiments with stepwise removal of the 5 ′-FS of 4.5SH RNA gene and replacing it with a vector polylinker, we discovered transcripts longer than 4.5SH RNA; this happened when the TATA-like box was removed completely (constructs −19 and −12 in Figure 1). Transcript elongation was also observed in case of simultaneous replacement of three nucleotides (−30, −28, −27) in the TATA-like box (Figure 4). Since the 4.5SH RNA gene has an effective transcriptional terminator (T_7_), it is highly unlikely that this elongation is due to impaired transcription termination. We believe that RNA lengthening occurs due to a shift of the transcription start site (TSS) into 5′-FS. Elongated transcripts were also observed in experiments on transient transfection of HeLa cells with B1 copies; such transcripts appeared when SINE copies were transcribed relatively weakly (Figure 7, copies 1, 2, 4, and 6). We believe that copies of B1 with −31/−24-boxes that do not fully meet the requirements for TATA-like boxes are not only transcribed more weakly, but also begin to transcribe from the sites located upstream. This is confirmed by normalization of the transcript length following the introduction of one or two substitutions into the −31/−24-box of B1_1, which improves its efficiency (Figure 8A). Replacing the −31/−24-box in copy B1_6 with GC-rich box not only reduces the level of transcription, but also increases the length of the RNA (Figure 8C). Interestingly, such transcript elongations were much less frequent in 4.5SI RNA gene and SINE B2.

The −40/−13 region of the yeast *SUP4 tRNATyr* gene was replaced with GC-rich sequence and then different regions of this new sequence were replaced with a TATA box, which led to a shift of TSS up to positions −11 or +8 [57]. The authors suggested that TBP, as a part of TFIIIB and in cooperation with TFIIIC, can find a TA-rich sequence and bind it. Due to the fact that TFIIIC subunit Tfc4 that directly interacts with TFIIIB, is able to stretch and contract, the position of the TBP binding site can vary within the range of 30 nucleotides. As a result, the TSS may shift in either direction, depending on where the TATA box is located. Presumably, the observed extension of 4.5SH RNA gene and SINE B1 transcripts in the absence of a sequence suitable for TFIIIB binding at position −31/−24 can be explained in accordance with this model. However, it remains not quite clear why elongated transcripts were found less frequently in experiments with the 4.5SI RNA gene and SINE B2 in similar constructs. Perhaps, the model [57] is not fully applicable to our data, since these authors transcribed the tRNA gene in the yeast cell-free system. In addition, it is likely that the clear tendency of the 4.5SH RNA gene and B1 SINE to TSS shifting, and a slight tendency to this among the 4.5SI RNA gene and SINE B2, is associated with a lower similarity of Box A to consensus in the first pair than in the second (see above).

### 3.4. Potential Regulatory Elements in 5′-Flanking Sequences of Genes with Type 2 pol III Promoters

The analysis of 5ʹ-FSs of 4.5SH RNA and 4.5SI RNA genes revealed regions similar to the transcription factor recognition sites, in addition to TATA-like boxes. In the case of 4.5SH RNA genes, these are the CRE (position −51/−44) and Sp1 (region −90/−60) sites. Interestingly, these sites, as well as the sequence of the TATA-like box of 4.5SH RNA genes, turned out to be very similar to the corresponding sites in the 5ʹ-FS of 7SL RNA genes (Figure 2A). Presumably, the 4.5SH RNA genes acquired these three types of regions independently of the 7SL RNA genes, and their resemblance is due to some common features in the regulation of their transcription. However, one should take into account the significant similarity of the nucleotide sequences of these genes, due to their evolutionary relationship. Quite probably, in rodents SINE pB1d10 evolved by retroposition of 7SL RNA, and one of its copies transformed into the 4.5SH RNA gene in the ancestor of mouse-like rodents (Myodonta) [28,43]. Therefore, one can suggest the 5ʹ-FS of 4.5SH RNA gene was obtained from one of the 7SL RNA genes as a result of homologous recombination. This could explain the resemblance of TATA-like boxes and the presence of CREB and Sp1 recognition sites at similar positions in 7SL RNA and 4.5SH RNA genes.

A sequence resembling the CRE site was found at position −54/−47 of the murine 4.5SI RNA genes *Mmu1ʹ* and *Mmu1*. However, in other 4.5SI RNA genes of mouse (Mmu), rat (Rno), and hamster (Cgr), there were no sites resembling the same CRE site (Figure 2B). But at a similar position (−51/−42) to the 4.5SI RNA genes from the genomes of mouse (Mmu2), rat (Rno2, Rno3), and hamster (Cgr1, Cgr2), a sequence identical to the C/EBP factor recognition site was found (Figure 2B). A recognition site for this factor had not been previously detected in 5ʹ-FSs of genes transcribed by pol III.

In addition to tRNA genes, there are only eight genes with pol III promoters of type 2 in mammals, including two human virus genes (Table 1). For this reason, the study of the 5′-FSs of the two of them (4.5SH and 4.5SI RNA) may be a significant contribution to elucidating the patterns of the structure of 5ʹ-FSs in genes with type 2 promoters. All of these genes have TATA-like boxes that are significantly different from each other, but usually these heptamers contain only two or three residues G or C (Table 1). In all genes, except BC200, at position −50 there is a CRE factor recognition site (Table 1). In some variants of 4.5SI RNA gene, a C/EBP recognition site is located at this region. Finally, Sp1 recognition sites are located at the −90/−60 region of the genes of four RNAs (7SL, 4.5SH, EBER 1, and EBER 2). (As we have found, depending on the species and variant of the 7SL RNA gene, the number of Sp1 sites can reach three, although there may not be any at all. Figure 2A). In general, structural diagram of 5′-FSs of this type genes includes a TATA-like box, a CRE site (in most cases), or C/EBP, and finally, in some cases, a Sp1 site.

We excluded tRNA genes from the analysis, because they do not contain the binding sites of transcription factors, such as CREB or Sp1. In addition, computer analysis of the 5′-FSs of mammalian tRNA genes did not reveal any uniform and conserved motifs [16,17]. To a large extent this applies to other animals, but not to plants in which tRNA genes possess TATA boxes. It is believed that during evolution, animal tRNA genes lost their TATA boxes, which was probably offset by the appearance of multiple splicing variants of two of the TFIIIB subunits, Bdp1, and Brf1 [17]. However, it is possible that the tRNA genes of mammals and other animals have TATA-like boxes that resemble those shown in the table; the variety of such boxes and small variations in localization do not allow them to be identified in statistical computer analysis of tRNA genes. Perhaps an individual analysis of mammalian tRNA genes would reveal TATA-like boxes. This analysis is difficult, due to the fact that the 5′-terminal sequences of the primary transcripts are deleted during tRNA maturation; therefore, the localization of TSS up to the nucleotide may remain unknown for most tRNA genes.

Using transfection of HeLa cells, we evaluated the effect of the recognition sites CRE and Sp1 in the 5ʹ-FS on transcription of 4.5SH RNA gene. It turned out that the CRE site strongly affects transcription: its replacement (deletion) reduced the efficacy of transcription by three times. On the contrary, the removal/replacement of the Sp1 site reduced the efficacy of transcription by no more than 28%, and this decrease was quite reliable only in the experiment with the jerboa gene. Similar data were obtained for 4.5SI RNA gene. Substitutions of CRE site in the 5ʹ-FS of *Mmu1′* gene and C/EBP site in the 5ʹ-FS of *Mmu2* gene reduced the transcription by 30% and 50%, respectively. The data obtained indicated that the contribution of the studied recognition sites of CREB, C/EBP, and Sp1 factors to the transcription efficiency of 4.5SH and 4.5SI RNA genes is relatively small. These values are comparable to those obtained by inactivation of the CRE sites in the 7SL RNA gene [21], as well as CRE and Sp1 sites in the EBER2 RNA viral gene [20]. Quite possibly, in other cells (for example, non-cancerous) or other cell growth conditions, the contribution of CREB, C/EBP, and Sp1 factors to the transcription efficiency of 4.5SH and 4.5SI RNA genes may happen to be significantly greater.

Unlike the genes presented in Table 1, SINE copies contain no recognition sites for the transcription factors ATF/CREB or C/EBP around position −50 in their 5′-FSs. It is known that SINEs are hardly transcribed by pol III in normal cells, which protects their genomes from the integration of new SINE copies [62,63,64]. The absence of binding sites for the above transcription factors in the upstream sequences of SINEs can be one of the main factors of their repression in normal cells. In such cells, pol III transcription is repressed by p53, which can bind to TBP in TFIIIB [65,66], and thus inhibit SINEs transcription. ATF/CREB was shown to bind its site in the upstream region of the 7SL RNA gene, and to prevent p53-induced repression of the gene transcription [65]. The same is likely true for other genes with pol III promoters presented in Table 1. One may presume that such a regulatory mechanism does not work in tumor cells with non-active (mutant) p53, which leads to the active pol III transcription of SINEs [62,64], and the emergence of their copies in new genomic loci. This scheme concerning the role of 5′-FSs in the transcription of SINEs and non-tRNA genes with type 2 pol III promoters requires further experimental confirmation.

## 4. Materials and Methods

### 4.1. Plasmid Constructs

The constructs containing murine genes of 4.5SH RNA [49] or 4.5SI RNA [30] with deletions or substitutions in the 5′-FSs were obtained by PCR. The products of amplification were purified using electrophoresis in 2% agarose gel with subsequent DNA isolation by Cleanup Mini columns (Evrogen, Russia). The obtained DNA fragments were cloned into pGEM-T plasmid (Promega, Madison, WI, USA). By colony PCR, the clones with the same insert orientation were picked: in such inserts, 5′-FS of the gene under study was adjacent to the polylinker region, which starts with *Spe I* site. Therefore, in all the constructs the same plasmid sequence was adjacent to the 5′-flanking gene region, which guaranteed the same influence of the upstream plasmid DNA on gene transcription in different clones. The possibility of such influence has been described in our earlier studies [47,49]. The clones with the deletions or substitutions of interest and without spontaneous mutations were grown, and the plasmids for subsequent transfection were isolated by NucleoBond PC 100 kit (Macherey-Nagel, Dylan, Germany).

The B1 copy from the first intron of α-fetoprotein gene and the B2 copy from the third intron of the *Cxcl 16* gene (this B2 copy was one of the first described under the name of Mm14 [23]) were chosen for study as the initial murine SINE sequences. Both these SINE copies have functional A and B boxes of the pol III promoter, and are capable of effective in vitro transcription [48,67]. Using UCSC Genome Browser, we searched for murine B1 and B2 copies that are the most similar to the abovementioned copies of these SINEs. By means of PCR with mouse genomic DNA, ten such B1 copies and eight B2 copies were amplified. The PCR products containing SINEs as well as 50–70 bp flanking sequences were cloned into pGEM-T plasmid as was described above for 4.5SH and 4.5SI genes. After the structure of the cloned DNA was confirmed by sequencing, another PCR round was performed. In this case, the reverse primer was complementary to the B1 or B2 region situated immediately downstream of box B. Following the PCR product cloning into pGEM-T, the constructs containing 5′-part of the SINE (mini-B1 or mini-B2) with the genomic 5′-FSs were obtained. In all the constructs, these 5′-FSs were adjacent to the pGEM-T polylinker region, starting with *Spe I* site. The changes to the 5′-FSs of the studied B1 and B2 copies were introduced by PCR and subsequent cloning of the amplified DNA fragments into pGEM-T. All B1 and B2-containing plasmids designed for transfection were isolated by NucleoBond PC 100 kit (Macherey-Nagel, Dylan, Germany).

### 4.2. Cell Transfection and Northern-Blot Analysis

HeLa cells (ATCC, CCL-2) were grown to 80%-confluent monolayer in Petri dishes (60 mm diameter), using DMEM with 10% fetal bovine serum. Cells on one dish were transfected by 5 μg of DNA of the plasmid construct, mixed with 10 μL of TurboFect reagent (Thermo Fisher Scientific, Waltham, MA, USA), according to the manufacturer’s protocol. Before adding TurboFect, the studied construct was mixed with the control plasmid containing shrew SINE SOR [68,69]; the quantity of the added control plasmid depended on the type of the sequence under study, and equaled 0.125, 0.5, 0.25, and 0.5 μg in the experiments with 4.5SH, 4.5SI genes, SINEs B1 and B2, respectively. In this work, we used human cells (HeLa) for transfection because, unlike any mouse cells, they do not contain endogenous 4.5SH and 4.5SI RNA, as well as B1 and B2 transcripts. Due to this, HeLa cells are rather suited for monitoring the transcription of these mouse genes and SINEs in them.

The cellular RNA was isolated in 20 h after transfection using guanidine-thiocyanate method [70]. RNA samples (10 μg) obtained as a result of each transfection were separated by electrophoresis in 6% polyacrylamide gel with 7M urea. Then RNA was transferred to Hybond-XL membrane (GE Healthcare, Chicago, IL, USA) using semi-dry electroblotting, and was hybridized with probes labeled by α[^32^P]dATP, Taq-polymerase, and reverse primers (Figure S1). The hybridization was performed incubating the membrane in the solution containing 50% formamide, 5×Denhardt solution, 4× SSC, 1% SDS, 0.1 mg/mL salmon DNA, and the labeled probe, at 42 °C overnight. The membrane was washed in 0.1× SSC with 0.1% SDS at 42 °C for 1 h and exposed to X-ray film. To quantify the radioactivity of the hybridization signals, the membrane was scanned in phosphorimager Cyclone (Packard). The hybridization signals of the 4.5SI and 4.5SH transcripts, as well as SINE B1 and SINE B2 transcripts, were normalized to the signals of SINE SOR transcripts.

## 5. Conclusions

**Figure 13:**
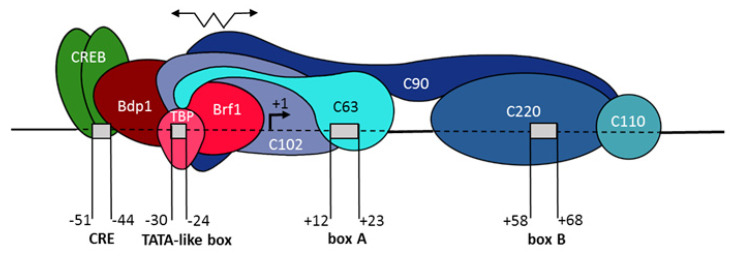
A complex of TFIIIB, TFIIIC, and CREB with a gene carrying a type 2 promoter for pol III. Factor binding sites in the gene and its 5′-FS are shown as boxes. The subunits of TFIIIB and TFIIIC are colored in different shades of red and blue, respectively. The transcription start site is marked as +1. The vertebrate tRNA genes and SINEs lack specific TATA-like boxes and CRE sites, but otherwise have a structure similar to that shown in the diagram. The zigzag arrow symbolizes the ability of the C63 and C102 subunits to stretch, which allows TFIIIB to bind to a more distant site in the 5′-FS of a SINE copy in case of high GC content at −31/−24. The scheme is based on a model for the interaction of TFIIIB and TFIIIC subunits with human tRNA genes (modified from [9] with permisson).

The scheme in Figure 13 illustrates the interaction of TFIIIB, TFIIIC, and CREB with the genes discussed above possessing boxes A and B, TATA-like boxes, and CRE. TFIIIC binds to boxes A and B, and then recruits TFIIIB, which comes close to the 5′-FS at the TATA-like box area. TFIIIB bends and unwinds DNA in the area and, through its subunit Brf1, recruits pol III, which transcribes the gene. Transcription of the 4.5SH RNA gene is particularly sensitive to the removal of a TATA-like box. The transcription factor CREB/ATF binds to its CRE recognition site (near position −50) and, by a yet unknown mechanism, stimulates the formation of a transcription complex. The same applies to the C/EBP factor for some variants of the 4.5SI RNA gene. SINE copies do not have CRE or C/EBP recognition sites, as well as particular TATA-like boxes. However, the function of the latter can be successfully performed by various nucleotide sequences located upstream the SINE copies at position −31/−24. If the sequence at this position has a high G + C content, SINE transcription is weak; moreover, in these cases, transcription can start from the site located upstream of the first SINE nucleotide. Presumably, TFIIIC subunits that bind TFIIIB are able to extend, allowing the latter to “find” a sequence that better matches the requirements for the TATA-like boxes than the DNA located at position −31/−24. Thus, the SINE copies in an unfavorable genomic environment can still be capable of transcribing.

## Figures and Tables

**Table 1:** The potential regulatory elements in 5′-flanking sequences of genes transcribed by pol III from type 2 promoters.

NcRNA (Gene) ^1^	TATA-like Box: Sequence ^2^, Position	−50 Elements: Name ^3^, Position, Sequences ^4^	−90/−60 Elements ^3^	References
Epstein–Barr virus: EBER 1 EBER 2	ATGTAGAC −28/−22 GTATAGAG −28/−22	**Atf**/**Cre** −51/−44 TGACGTAG TGACGTAG	**Sp1** −65/−60 CCGCCC CCCGCC	[20,58]
Adenovirus VAI	CTAGACCG −30/−24	**Atf**/**Cre** −38/−31 TGACGCTC		[59,60]
7SL-1 (Hs) 7SL-3 (Hs)	TTCTAGTA −29/−23 TTCTAGTG −30/−24	**Atf**/**Cre** −50/−43 TGACGTCA TGACGTAA	Sp1 GGGCGG CCCGCC	[21], this study
vt	TCAAGAAA −30/−24	Atf/Cre −53/−46 TGACGTAG		[22]
BC200/G22	CTATGAAA −32/−26		about −80/−70 unidentified	[61]
4.5SH	TTCAAGTA −30/−24	**Atf**/**Cre** −51/−44 TGTCGTCA	**Sp1** Region: −87 to −60 GGGCGG ^5^	This study
4.5SI *Mmu1ʹ* 4.5SI *Mmu2*	CTACATGA −30/−24 CTACTTCA −30/−24	**Atf**/**Cre** −54/−47 TGACGTAC **C**/**EBP** −51/−42 GTTGCGCAAC		This study and [47]

^1^ The data presented apply to two human 7SL RNA genes and two murine 4.5SI RNA genes. ^2^ Nucleotides constituting the TATA-like box itself are underlined. ^3^ The names of transcription factors recognition sites are given in bold, if the effect of these sites on transcription was checked experimentally. ^4^ The nucleotides corresponding to the canonical sequence of the transcription factor recognition site are underlined. ^5^ Here, the typical GC-box from 5′-FS of rat and jerboa gene is shown; the murine site is presented by GGGCCG hexamer.

## Supplementary Materials

Supplementary materials can be found at https://www.mdpi.com/1422-0067/21/10/3706/s1.

## Abbreviations

5′-FS	5′-flanking sequence
ATF	activating transcription factor
Bdp1	B double prime 1 factor
Brf	TFIIIB-related factor
C/EBP	CCAAT-enhancer binding protein
CRE	cAMP-response element
CREB	cAMP-response element binding protein
DSE	distal sequence element
EBER	Epstein-Barr virus-encoded RNA
pol III	RNA polymerase III
PSE	proximal sequence element
SINE	short interspersed element
TBP	TATA box binding protein
TFIII	transcription factor for pol III
TSS	transcription start site
vtRNA	vault RNA
