# Supplementary material for: TATA-Like Boxes in RNA Polymerase III Promoters: Requirements for Nucleotide Sequences
Source: Int J Mol Sci. 2020 May 25;21(10):3706. doi: 10.3390/ijms21103706 (PMC7279448; doi:10.3390/ijms21103706)
Supplement: Supplementary file 1 [file ijms-21-03706-s001.pdf]

4.5SH RNA gene
GCCGGTAGTGGTGGCGCACGCCGGTAGGATTTGCTGAAGGAGGCAGAGGCAGGAGGATCACGAGTTC
GAGGCCAGCCTGGGCTACACATTTTTTT

4.5SI RNA gene
GGGCTGGAGAGATGGCTCAGCCGTTAAAGGCTAGGCTCACAACCAAAAATATAAGAGTTCGGTTCCCA
GCACCCACGGCTGTCTCTCCAGCCACCTTTTTT

B1 SINE from the1-st intron of AFP gene (mm10_rmsk_B1_Mus2 range=chr5:90490962-90491107)
GCCGGGTGTGGTGGCGCACACCTTTAATCCCAGCACTCGGGAGGCAGAGGCAGGCGGATTTCTGAGTT
CGAGGCCAGCCTGGTCTACAAAGTGAGCTCCAGGACAGCCAGGGCTACACAGAGAAACCCTGTCTCAA
AAAAAAAAAAAAAAAAAAAAgatacctggagatgcaaatatgggaatttttaa

B2 SINE, clone Mm14, >mm10_rmsk_B2_Mm2 range=chr11:70458328-70458510
GGGCTGGAGAGATGGCTCAGCGGTTAAGAGCACTGACTGCTCTTCCGAAGGTCCTGAGTTCAATTCCCG
GCAACCACATGGTGGCTCACAACCATCCGTAATGAGATCTGGTGCCCTCTTCTGGAGTGTCTGAGGACA
GCTACAGTGTACTTACATATAATAAATAAATCAACAAATCTTTT

Fig. S1. Nucleotide sequences of 4.5SH RNA and 4.5SI RNA genes as well as B1 and B2 copies used to prepare hybridization probes (underlined). Probes were generated by PCR. Forward primer sequences are highlighted in green and sequences complementary to reverse primers are shown in red letters. The probes were labeled by PCR (20 cycles) with α[^{32}P]dATP and the reverse primers. B2 SINE clone Mm14 was described in Krayev *et al.*, 1982.

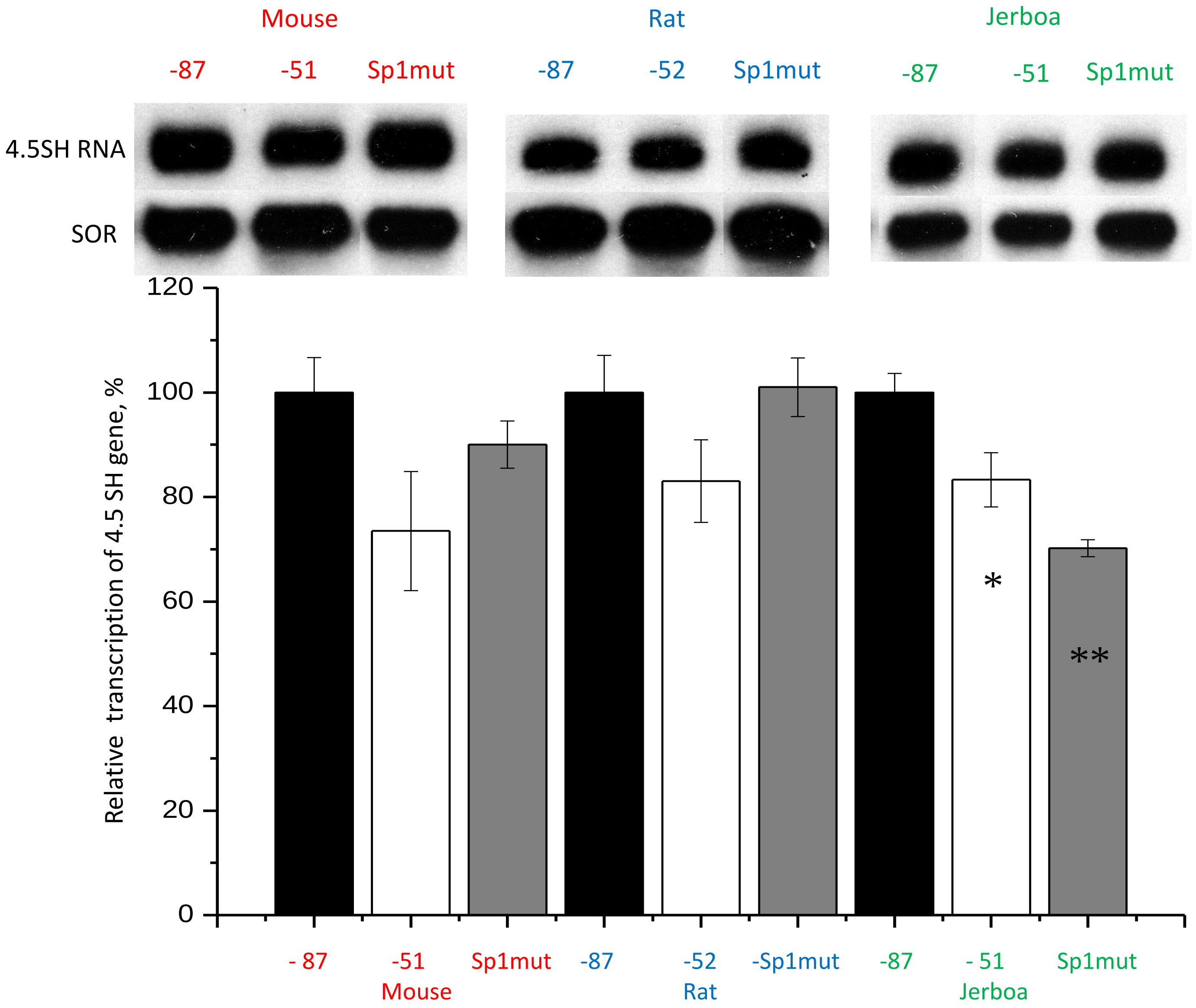

Fig. S2. Effect of Sp1 site deletions and substitutions in 4.5SH RNA genes of mouse, rat, and jerboa on their transcription. The constructs with deletion up to position -87 have Sp1 sites, while the constructs with deletions up to position -51 or -52 (rat) lack them (see Fig. 2). In Sp1mut constructs, Sp1 sites were replaced with the AAATAA hexamer and thus inactivated (error bars, SD, N=3).

```
          -50       -40       -30       -20       -10        -1
-54 WT   GCGGTTGCGCAACGCCTAACTTGCTACTTCAGGGAAAGAATACAGGGCGTCGTC
-41      GCG----------GCCTAACTTGCTACTTCAGGGAAAGAATACAGGGCGTCGTC
-54 mut GCGTGGAGTTCGTGCCTAACTTGCTACTTCAGGGAAAGAATACAGGGCGTCGTC
```

A

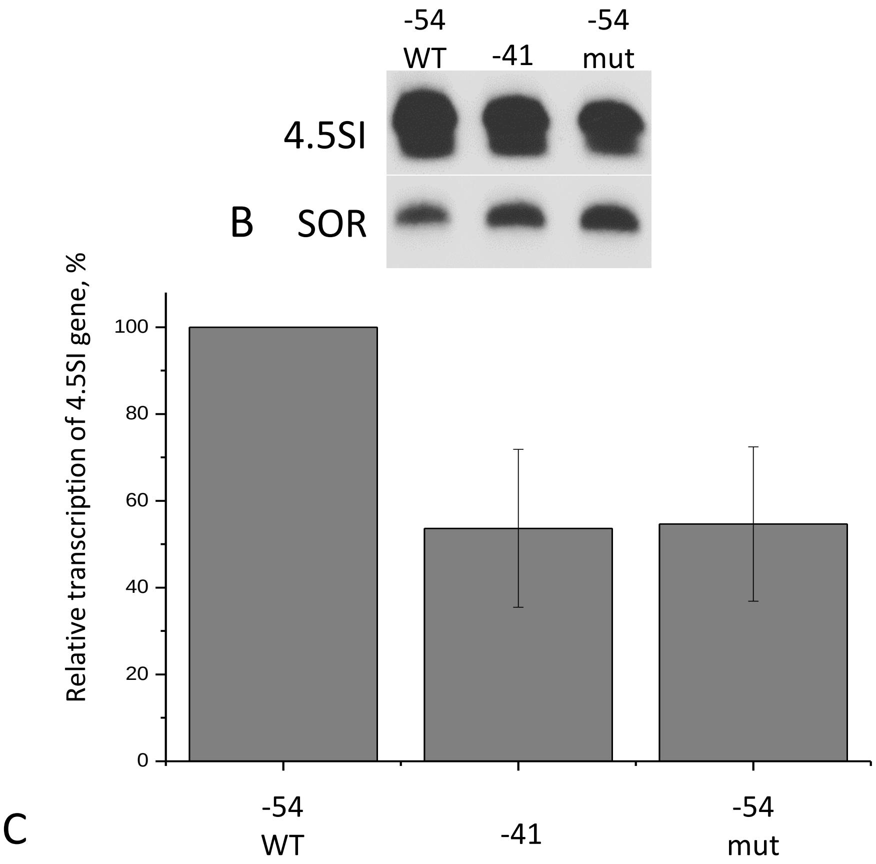

Fig. S3. Effect of C/EBP site (-51/-42) in 5’-FS of 4.5SI RNA gene Mmu2 on transcription. (**A**) 5’-FSs in three constructs used i[illegible] transfection experiments. The deleted nucleotides are indicated by dashes; the replaced nucleotides are given in bold. (**B**) The transcripts of the 4.5SI RNA gene detected in the transfected cells by Northern hybridization. SOR SINE was used as control and to normalize Northern hybridization data. (**C**) Quantitative analysis of the Northern hybridization data (error bars, SD, N=3).

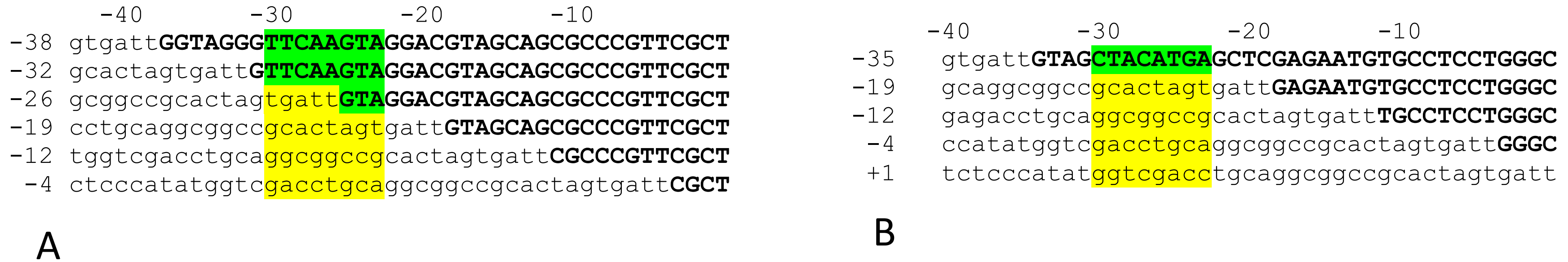

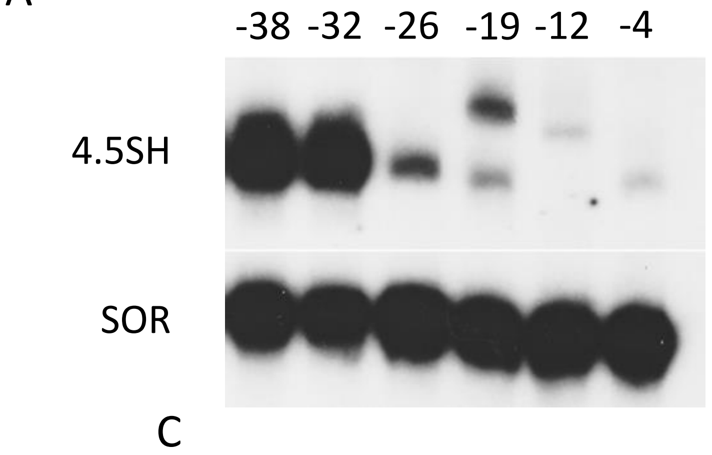

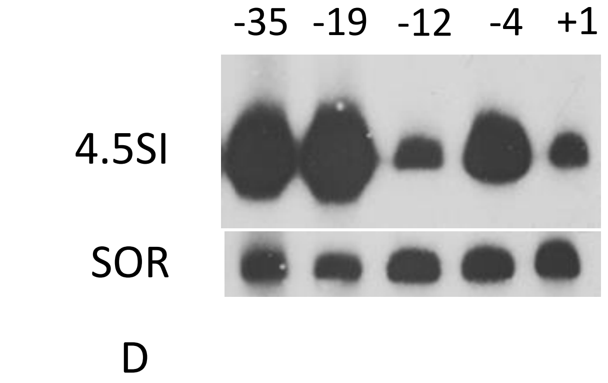

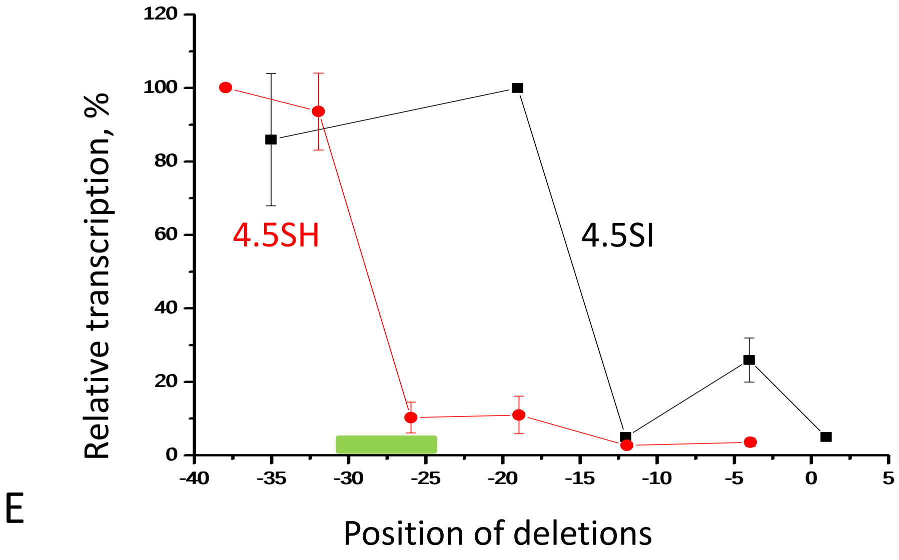

Fig. S4. Comparison of effect of deletions in 5′-FSs of murine 4.5SH RNA and 4.5SI RNA genes on their transcription. 5′-FSs of 4.5SH RNA (**A**) and 4.5SI RNA (**B**) genes in the constructs used for transfection. The murine and plasmid polylinker sequences are given in uppercase and lowercase, respectively. The murine boxes -31/-24 are marked in green; the polylinker nucleotides replacing the box are highlighted with yellow. The constructs are named according to the number of the first nucleotide in the upstream murine sequence (indicated at the left). The transcripts of genes 4.5SH RNA (**C**) and 4.5SI RNA (**D**) detected by Northern hybridization in transfected cells (4.5SI RNA data are from Gogolevskaya et al., 2018). (**E**) Graphs of transcription rate versus the length of the remaining murine 5′-FS of genes 4.5SH RNA and 4.5SI RNA (error bars, SD, N=3). The green rectangle shows the -31/-24 box position.
